# Multifunctional Upconversion Nanoparticles Transforming Photoacoustic Imaging: A Review

**DOI:** 10.3390/nano15141074

**Published:** 2025-07-10

**Authors:** Yuqian Zhang, Zerui Li, Ziqing Du, Jianming Pan, Yanan Huang

**Affiliations:** 1 Nepean Hospital, Kingswood, NSW 2747, Australia; 2 School of Chemistry and Chemical Engineering, Jiangsu University, Zhenjiang 212013, China; 3 School of Materials Science and Engineering, University of New South Wales, Sydney, NSW 2052, Australia; 4 School of Natural Sciences, Macquarie University, Sydney, NSW 2109, Australia

**Keywords:** photoacoustic imaging, lanthanide ions doped nanoparticles, NIR absorption, upconversion nanoparticles, contrast agents

## Abstract

Photoacoustic imaging (PAI) merges the high spatial resolution of optical methods with the deep tissue penetration provided by ultrasound, making it a valuable tool in biomedical imaging. In recent years, a diverse array of photoacoustic contrast agents, spanning both organic and inorganic materials, has been developed. Among them, upconversion nanoparticles (UCNPs) stand out as promising candidates due to their unique optical features, tunable absorption in the near-infrared I (NIR-I, 750–1350 nm) region, and strong potential for both imaging and treatment-related uses. This review discusses the growing significance of UCNPs in the field of PAI, focusing on their structural characteristics, strengths, and existing challenges. Then, we talk about an up-to-date account of the current literature on the use of UCNPs as contrast agents for PAI. Lastly, we discuss the challenges and perspectives of UCNPs as a contrast agent for PAI in preclinical research and clinical diagnosis.

## 1. Introduction

Numerous non-invasive imaging technologies, for instance, magnetic resonance imaging (MRI), computed tomography (CT), positron emission tomography (PET), and ultrasound (US) [1,2,3,4,5,6], have been extensively applied in the biomedical field. Among these, optical imaging stands out as a convenient method for real-time visualization and quantification of biological processes in living organisms. It avoids the use of ionizing radiation, making it especially attractive for both clinical applications and biological studies [7]. In recent developments, hybrid imaging techniques like X-ray excited optical luminescence (XEOL) combined with PET have been introduced [8], leveraging multiple physical principles to enhance imaging resolution, depth, speed, and three-dimensional reconstruction beyond what single modalities can achieve. Despite these advancements, conventional optical imaging alone has struggled to deliver high-resolution images at centimeter-level depths—until the emergence of photoacoustic imaging (PAI) [9].

**Figure 1:**
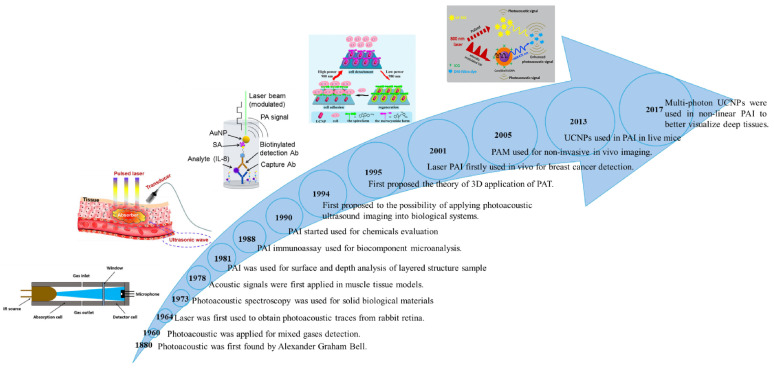
Timeline of photoacoustic imaging development. Tracing back to Alexander Graham Bell’s early discovery of photoacoustic principles to its eventual application in clinical settings, the technology has evolved over several decades [13,34,35,36,37,38,39,40,41,42,43,44,45].

**Figure 2:**
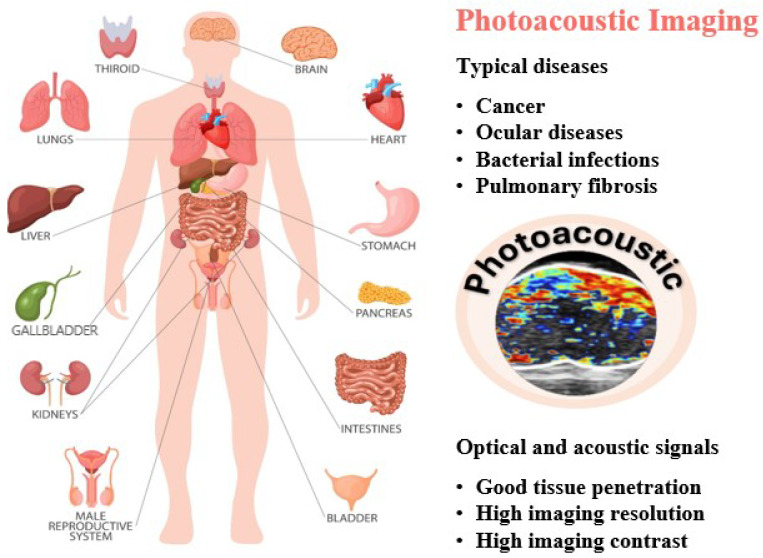
Scheme of photoacoustic imaging technology in precise disease detection and diagnosis.

PAI, a relatively recent innovation, synergizes the strengths of optical and ultrasound imaging to enable highly sensitive visualization of optical probes [10,11,12]. Over the past decade, PAI has gained prominence as a technique that relies on laser-induced ultrasound generation. It was in 1880 that Alexander Graham Bell first noted the photoacoustic (PA) response, observing sound waves generated by light absorption [13]. PAI has gained traction since the 1990s because of progress in laser technology and ultrasound detectors (Figure 1) [14]. PAI offers impressive spatial (around 100 µm) and temporal (approximately 1 ms) resolution, constrained primarily by the specifications of the US transducer. Concerning traditional imaging, PAI employs the optical wavelengths in the near-infrared (NIR, 700–1500 nm), achieving penetration depths up to several centimeters. Versus conventional imaging modalities [15,16,17,18,19,20,21,22,23,24,25,26,27], PAI utilizes near-infrared (NIR, 700–1500 nm) light to achieve imaging depths reaching several centimeters [28]. One of the main benefits of PAI is its capacity to provide detailed structural, functional, and molecular information about biological tissues using non-ionizing energy sources. Through the integration of optical stimulation and ultrasound signal acquisition, this technique allows for real-time imaging capabilities [29]. PAI emerges as a robust imaging technique capable of offering detailed anatomical and physiological information, particularly valuable for the early diagnosis of certain tumors (Figure 2) [30,31,32,33].

In the realm of PAI, a key area of ongoing research lies in the design and refinement of nanostructured imaging enhancers that offer improved contrast-to-noise ratios, minimal toxicity, and efficient clearance from the bloodstream [28]. To overcome these challenges and improve the sensitivity and depth of imaging, PAI contrast enhancement agents are required [46,47]. The strength and high resolution of PAI have been demonstrated using extrinsic contrast agents such as fluorescent organic dyes [48,49], dye-loaded nanoparticles [50], carbon nanotubes [51,52,53], semiconductor quantum dots (QDs) [54,55,56], metal complexes [57,58,59], and fluorescent proteins [60,61]. These agents have been utilized for multiple types of preclinical PAI applications, including (1) subcellular imaging of organelles like mitochondria and melanosomes in vitro; (2) in vivo tracking of individual cells such as erythrocytes alongside malignant melanocytes; (3) visualizing vascular architectures, neovascularization, and the lymphatic system; (4) evaluating physiological parameters including hemoglobin oxygenation, blood flow dynamics, and metabolic activity within microvasculature; (5) assessing brain activity; and (6) monitoring drug delivery processes, therapeutic outcomes, targeted molecular interactions, and gene expression patterns [62,63,64,65,66,67].

In recent decades, upconversion nanoparticles (UCNPs) have emerged as innovative contrast agents across various biomedical imaging modalities [68,69,70]. These nanoparticles have the unique ability to alter low-energy light, typically in the near-infrared (NIR) region (wavelengths above 700 nm) [71], higher-energy emissions such as ultraviolet (UV, <400 nm), and visible light (400–700 nm) [72,73]. This upconversion (UC) process is based on nonlinear optical phenomena, where multiple photons are absorbed in succession via long-lived intermediate energy states, ultimately producing anti-Stokes emission—light with emission occurring at a shorter wavelength relative to the excitation beam, which was first recognized and formulated in the mid-1960s [74]. With advancements into nanomaterials that display upconversion behavior, their application in biomedical imaging has progressed rapidly. The specific advantages of UCNPs include: (1) the tunable NIR spectral absorbance in the “optical transmission window” of biological tissues (700–1000 nm); (2) no background autofluorescence unlike FL imaging probes; (3) applicability in multimodal imaging such as MRI, CT, PAI, and so on, and ability to enable photo-therapy [44,75,76,77,78,79]; (4) tissue-specific targeting through surface modification; (5) high in vitro and in vivo photostability; and (6) high biocompatibility. All of these merits render UCNPs more appealing than conventional fluorescence nanomaterials for bioimaging [80,81,82]. Therefore, UCNPs have been utilized as a modern iteration of a fluorescent nanoprobe for fluorescent optical imaging [83,84,85], holding great promise to overcome the innate shortcomings associated with dye molecules [86,87,88,89,90], fluorescent proteins, and quantum dots (QDs) that need to be excited with high-energy incident light, such as UV or visible light [91,92,93,94,95,96,97,98,99].

UCNPs as nanoprobe/CA in imaging have been broadly covered in multiple reviews in the past [59,100,101,102,103,104,105,106,107,108,109,110,111,112,113,114,115,116]. However, UCNPs as contrast agents for PAI have not yet been dedicatedly summarized and analyzed. With their growing popularity as PAI contrast agents, there is a critical need to summarize the progress of this field in a critical review. Therefore, we systematically outline the recent progress on the use of UCNPs for PAI accounting for their fundamental properties that make UCNPs perfect for PAI agents. We also focus on current breakthroughs in UCNP-based PAI, which is intended to drive innovation in this domain and stimulate further curiosity from fellow researchers. At last, we highlight the remaining challenges and speculate for the prospective real-world use of UCNPs in biomedical imaging technologies.

## 2. Working Principle of PAI

### 2.1. Physical Underpinnings of PAI

**Figure 3:**
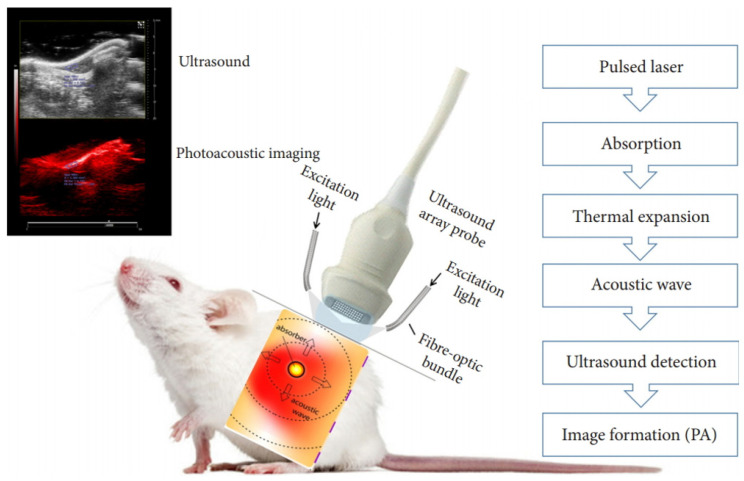
Fundamental mechanism of photoacoustic imaging. Absorbed light elevates local temperature, triggering thermoelastic expansion that generates US pressure waves through adjacent tissue. These waves are captured by US transducers, and image reconstruction is performed using their intensity and arrival time. Adapted with permission from Ref. [126].

PAI is a hybrid biomedical imaging technology that can achieve real-time and high-resolution in vivo tissue microanatomy [117]. PAI merges high-contrast optical excitation with strong temporal resolution (<100 µm) and high penetration depth of US imaging [118]. PAI typically uses NIR (750 to 1800 nm) and visible light (400 to 750 nm) to excite acoustic vibrations [119]. The photoacoustic effect involves three main stages: first, the target material absorbs incident photons, leading to a temperature rise; second, this thermal energy causes localized pressure elevation through thermoelastic expansion; and third, the resulting pressure disturbance propagates as acoustic waves via elastic interactions in the surrounding medium [120]. For effective generation of these acoustic signals, the thermal expansion must vary with time. This is commonly accomplished using a nanosecond pulsed laser (1–100 ns) or an intensity-modulated continuous-wave (CW) laser to illuminate the light-absorbing material [121,122]. Pulsed lasers are typically favored since they offer a higher signal-to-noise ratio than continuous-wave lasers at the same optical exposure and beam strength levels [123]. Additionally, the short pulse duration is typically less than the thermal and stress confinement times of the absorber, allowing thermal diffusion and volumetric expansion effects to be disregarded during excitation [124]. In biological tissues, photons interacting with cellular components predominantly undergo elastic scattering. When these scattered photons are absorbed by molecules, rapid thermoelastic expansion occurs, producing broadband ultrasonic waves in the surrounding tissue [125]. These acoustic signals are captured by piezoelectric transducer arrays, and the image is reconstructed by analyzing the amplitude and arrival time of the signals, enabling visualization of the original photoacoustic pressure distribution. A conceptual diagram of this mechanism is shown in Figure 3.

In general, upon absorption of photons by a tissue, the intrinsic UV–Vis chromophore is elevated to an excited state, followed by consequent chains of photon events in the visible and NIR region. In PA, due to NIR absorption capabilities of the chromophore, photon energy is transformed to heat energy via vibrational/collisional relaxation. This localized heating leads to increase in the local pressure. The initial local pressure rises or PA amplitude of waves ($P_{0}$) can be calculated using Equation (1).

(1) $$P_{0}=\Gamma H$$

In this context, H represents the optical energy or heat absorbed per unit tissue volume. The resulting pressure rise is generally considered to be linearly proportional. Photoacoustic signal efficiency generation largely relies on the Grüneisen parameter Γ, a dimensionless thermodynamic factor that is sensitive to temperature and differs among various absorbing materials. The absorbed energy density is obtained using Equation (2).

(2) $$H=\eta_{th}\mu_{a}F$$

Here, $\eta_{th}$ denotes the thermal conversion efficiency, which reflects the proportion of nonradiative energy dissipation after laser excitation (essentially equal to one subtracted by the fluorescence quantum yield) [124,127]. Since Γ is temperature dependent, influenced by temperature, PAI can serve as a tool for thermal monitoring [128]. The optical absorption coefficient $\mu_{a}$ (cm^−1^) reflects the concentration of the absorbing chromophore, and *F* denotes the local optical fluence (J·m^−2^). Collectively, these parameters define the total optical energy absorbed or deposited in the tissue [120,127,129]. Thus, combining Equations (1) and (2), PA amplitude of waves ($P_{0}$) can be determined as represented in Equation (3)

(3) $$P_{0}=\Gamma \eta_{th}\mu_{a}F$$

The main source of contrast is provided by variations in optical properties, absorption, and scattering by the sample. Among all, absorption tends to be the governing factor for contrast in PA imaging. In tissues where the temperature is high and light is well absorbed, the PA images show an optimal signal-to-noise ratio (SNR). Photoacoustic imaging (PAI) enables visualization of both anatomical and functional features—such as hemoglobin oxygen saturation and metabolic activity—either with or in the absence of external contrast agents [130], thus making this technique suitable for anatomical imaging.

### 2.2. PAI Implementation: Tomography and Microscopy

**Figure 4:**
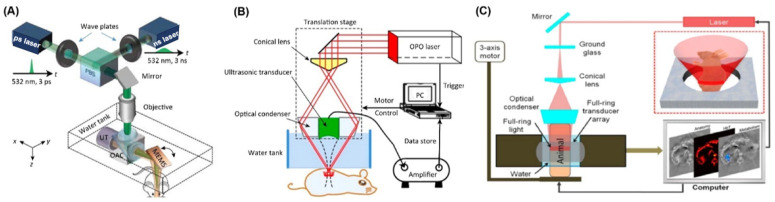
Multiscale PA imaging. (**A**) Schematic of OR-PAM, where fast scanning is attained by a MEMS mirror [144]. (**B**) Schematic of AR-PAM [144]. (**C**) Schematic of PACT [145]. Abbreviations: PA, photoacoustic; OR-PAM, optical-resolution PAM; MEMS, microelectromechanical scanner; OAC, optical-acoustic combiner; AR-PAM, acoustic-resolution PAM; UT, ultrasonic transducer. Reprinted with permission from Refs. [144,145].

According to the mechanism of formation of images, PAI systems are broadly categorized into two types: (i) raster-scanning-based photoacoustic microscopy (PAM) [67,131] and (ii) ultrasound array-based photoacoustic computed tomography (PACT) [132], as depicted in Figure 4. Photoacoustic imaging (PAI) systems can function in either reflection (backward) or transmission (forward) modes [133]. In the reflection mode, the laser source and US transducer are positioned on the identical side of the specimen and oriented perpendicular to it, similar to the arrangement in B-mode ultrasound imaging. In contrast, the transmission mode places the source and detector positioned on opposing sides of the sample, a setup typically used for thin tissue slices or ex vivo samples because of the limited light penetration in thicker tissues.

PAM inherits the features of PAI and is designed for in vivo imaging. PAM offers several benefits surpassing classical optical microscopy approaches, such as deeper imaging depth, highly sensitive structural and functional details, and not requiring optical sectioning to obtain the 3D image. PAM operates in either reflection or transmission mode and generally employs a focused single-element US transducer, paired with mechanical or optical scanning methods for image acquisition. In this configuration, the alignment of optical excitation and acoustic detection into dual focal points enhances overall sensitivity [134]. A single laser pulse produces a one-dimensional ultrasound signal along the axial direction of the laser beam. As the system scans laterally across the sample, these signals are collected point by point to reconstruct a three-dimensional image. The axial resolution is governed by the travel time of the acoustic wave, whereas the transverse resolution is influenced by the spacing between the optical and acoustic foci [134].

In the optical ballistic regime, the lateral resolution of photoacoustic microscopy (PAM) has been significantly improved through tight optical focusing, a technique known as optical-resolution photoacoustic microscopy (OR-PAM). This approach achieves high lateral resolution by relying on precise optical focus, enabling detailed in vivo imaging of individual cells and microvascular structures, like those found in murine auricular vessels. Furthermore, by harnessing nonlinear optical effects, the resolution of PAM has been pushed beyond the conventional optical diffraction limit, resulting in super-resolution imaging—termed photoacoustic nanoscopy (PAN)—which has made it possible to visualize structures as small as single mitochondria in vivo [135]. On the other hand, when acoustic focusing is applied within the optical diffusive regime, a method known as acoustic-resolution photoacoustic macroscopy (AR-PAMac) allows for complete anatomical imaging of small laboratory animals [136]. A notable benefit of this technique is its avoidance of complex reconstruction algorithms, thus minimizing image artifacts. Nonetheless, the reliance on raster scanning in PAM inherently limits the speed at which images can be acquired. To address this challenge, a new waterproof MEMS scanner (weighing 162 g with a diameter of 17 mm) has been created for OR-PAM [137,138]. These miniaturizations include mechanical scanning using a voice-coil motor (~15 Hz frame rate) [139], optical scanning using Galvo mirrors (~2 Hz frame rate) [140], and hybrid scanning through mechanical scanning on one axis and optical scanning on the other axis (~6 Hz frame rate) [141].

PACT is an evolving potent hybrid optical imaging modality utilizing both ultrasonic resolution and optical absorption contrast, while all the essential features are greatly scalable. PAT is extensively applied in research, with several commercial systems already available. In focused-scanning PAT, a focused ultrasound transducer physically scans across the target area. In contrast, array-based PACT typically utilizes transducer arrays arranged in linear, arc-shaped, or circular geometries [142]. A key benefit of this approach is its capability to deliver real-time imaging. Compared to PAM, PACT systems are more readily compatible with standard ultrasound platforms, making them more feasible for clinical integration and commercial development [143]. Typically, PACT offers spatial resolutions ranging from 0.1 to 1.0 mm, while maintaining the capability to image several centimeters deep into soft tissue. Additionally, a practical strength of this tomographic technique is that transducer arrays—such as linear probes—can be used in a handheld format, similar to conventional ultrasound devices.

## 3. Contrast Agent for PAI

### 3.1. Endogenous PAI—Intrinsic Chromophores

**Figure 5:**
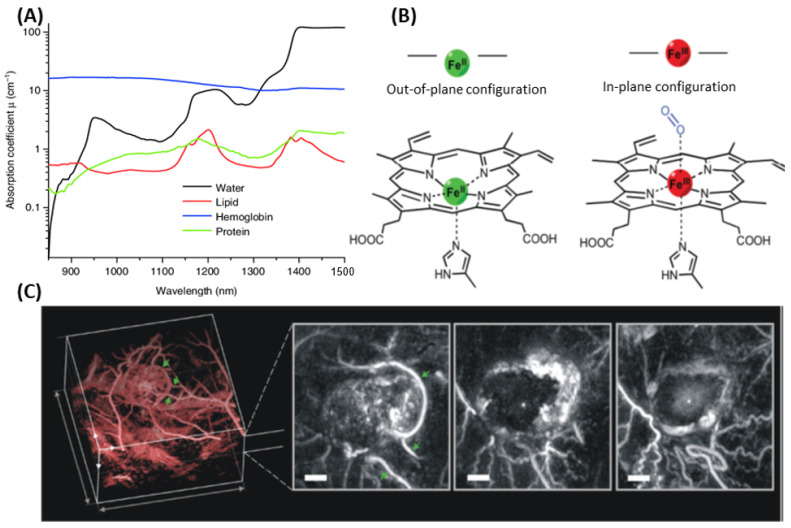
Photoacoustic imaging of intrinsic chromophores. (**A**) Absorption profiles of major endogenous molecules: hemoglobin (blue, 150 g/L), water (black, ~80% tissue volume) [166]; lipid, red line [167,168] (20% by volume in tissue); and protein, green line. Data from http://omlc.ogi.edu/spectra (access on 1 May 2025). (**B**) The heme moiety, an iron-bound aromatic porphyrin, undergoes structural changes upon oxygenation, leading to distinct spectral shifts. (**C**) PAI at 640 nm visualizes tumor vasculature before and 24 h after administration of the vascular disrupting agent OXi4503. Repeated vascular features in the skin are marked with green arrows. Scale bars: 1 mm. Figure 5 reprinted with permission from Ref. [169].

Endogenous photoacoustic imaging (PAI) captures both anatomical and functional data by detecting light absorption from intrinsic tissue components such as hemoglobin, lipids, water, and proteins (Figure 5A). Hemoglobin, responsible for oxygen transport, experiences structural and electronic alterations upon binding oxygen (Figure 5B), leading to distinct spectral shifts. These variations allow for the quantification of total hemoglobin levels and oxygen saturation, which are critical for analyzing tumor vascularization (Figure 5C) [146,147]. PAI can also be utilized to map lipid distribution in vivo [148], utilizing absorption peaks at 930 nm and 1210 nm, attributed to C-H bond overtones, while water content is mapped via its peak absorption near 975 nm [149].

### 3.2. Exogenous PAI—Key Factors Governing Contrast Agent Design

For effective molecular imaging, a contrast agent should consist of two key components: a signaling molecule responsible for generating the imaging signal, and a targeting unit that guides the agent to specific biological structures or processes for precise localization [150,151].

Nanoparticles used in photoacoustic imaging typically share several key features: (1) strong optical absorption in the near-infrared (NIR) window (usually 650–950 nm), which ensures deeper tissue penetration and efficient photoacoustic signal generation [152]; (2) high photothermal conversion efficiency to generate detectable acoustic waves upon pulsed laser excitation [153]; (3) good biocompatibility and stability under physiological conditions [154]; and (4) appropriate size and surface chemistry for prolonged circulation and targeted delivery [155]. These shared properties enable various types of nanoparticles—such as gold nanostructures, semiconducting polymers, carbon-based materials, and organic dyes—to serve as effective photoacoustic contrast agents.

(i) *Photophysical properties*

An optimal signaling molecule for photoacoustic imaging should possess several key features. First, a high molar extinction coefficient is essential for efficient light absorption and strong photoacoustic (PA) signal generation. While a distinct and narrow absorption peak can aid signal isolation under controlled conditions, its effectiveness in vivo is limited due to the broad absorption spectra of endogenous chromophores (e.g., hemoglobin, melanin) and tissue scattering, which compromise spectral specificity and introduce signal cross-talk. As such, relying solely on spectral unmixing is insufficient for accurate in vivo imaging. To overcome these limitations, alternative detection strategies have emerged.

One such approach is the use of radiometric photoacoustic imaging, in which dual-wavelength or dual-emission UCNP systems (e.g., Tm^3+^/Er^3+^- or Yb^3+^/Nd^3+^-doped nanocrystals) are employed to provide internal calibration [156,157,158,159], thereby reducing variability from probe concentration and environmental effects. Time-gated or lifetime-based detection methods also show promise, leveraging the long luminescence lifetimes of UCNPs to distinguish signal from tissue background and improve temporal resolution [160,161,162].

Furthermore, photo-switchable probes, such as UCNPs coupled with light-activated absorbers (e.g., spiropyrans), can create an on/off contrast mechanism, enhancing signal discrimination [157,163]. Similarly, stimuli-responsive systems—including thermoresponsive or pH-sensitive UCNP composites—enable signal activation only under specific physiological conditions, increasing target specificity and reducing background interference [164,165].

Ideally, the absorption of these constructs should fall within the near-infrared (NIR) window (620–950 nm) to maximize tissue penetration (Figure 5A). In addition, strong photostability is necessary to preserve signal over time, while a low fluorescence quantum yield is preferred to favor nonradiative decay and promote heat generation. Ultimately, the contrast agent must efficiently convert absorbed energy into localized thermal expansion to produce reliable and high-contrast acoustic signals, suitable for deep-tissue, high-resolution biomedical imaging.

(ii) *Biological properties*

Targeted contrast agents need to overcome circulatory and cellular barriers to effectively reach their specific target sites. Upon entering the bloodstream, these agents encounter various biomolecules, which may adsorb onto their surfaces [170,171,172]. This surface adsorption can influence immune recognition and interfere with target specificity, thereby complicating data interpretation and requiring thorough characterization [171,173]. To effectively extravasate from blood vessels into surrounding tissues, contrast agents generally need to be smaller than 10 nm. However, in oncology-focused uses, nanoparticles as large as 100 nm have been shown to traverse the abnormally permeable vasculature typically found in tumors [174]. After reaching the tissue interstitium, the agent may cross cellular barriers either via active targeting—such as binding to cell surface receptors [175], transporters [176], or metabolic enzymes [177]—or through passive mechanisms like diffusion or endocytosis.

### 3.3. UCNPs as Contrast Agent for PAI

(i) ***Synthesis and Functionalization of UCNPs***

Long before UCNPs emerged, upconversion (UC) phenomena had already been demonstrated in various bulk materials using diverse host and lanthanide (Ln^3+^) ion combinations. However, only a limited number of these combinations are viable in colloidal nanocrystals due to synthesis challenges and reduced emission efficiency at the nanoscale. Fluoride-based lattices like NaYF_4_ are commonly employed as UCNP hosts due to their low phonon energy (~350 cm^−1^), high optical transparency, and ability to form well-crystallized structures at relatively low synthesis temperatures. Various synthetic methods—including thermal decomposition, hydrothermal synthesis, sol-gel processing, coprecipitation, and ionic liquid-assisted techniques—have been explored to produce high-quality lanthanide-doped NaYF_4_ UCNPs with precise control over composition, crystallinity, and morphology (Table 1). Among these methods, thermolysis [178] and hydrothermal synthesis [179] are most frequently employed, as they enable fine control over the phase, morphology, size, and stoichiometry of both core and core–shell UCNPs.

**Figure 6:**
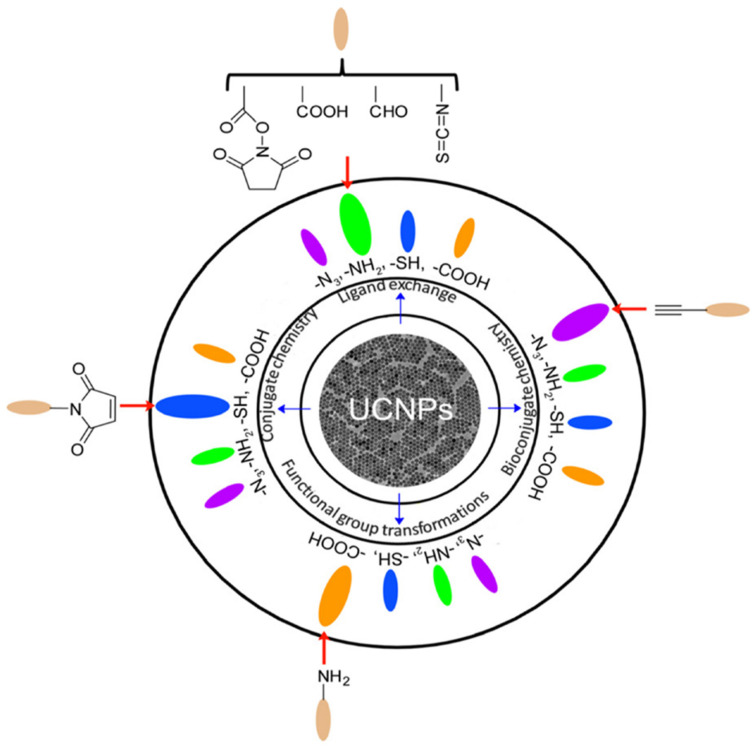
Commonly used methods of functionalizing UCNPs. Reproduced with permission from Ref. [187].

To obtain UCNPs with high crystallinity and uniform morphology, the above-mentioned methods are typically conducted in high-boiling-point non-aqueous solvents alongside one or two long-chain ligands. Consequently, the resulting UCNPs are usually coated with hydrophobic ligands (e.g., oleic acid), necessitating subsequent surface modification to render them hydrophilic and enable functionalization (Figure 6) [187]. These approaches can be broadly classified into three main categories: hydrophilic modification, bioconjugation, and functionalization with hybrid materials. Common approaches include the use of (1) acidic ligands [188], (2) polymers [189], or (3) chelating agents [190], all of which typically contain hydrophilic functional groups such as hydroxyls, amines, or carboxylic acids. A comprehensive overview of these modification techniques is provided in the recent review by Chen et al. [191].

(ii) ***Dopant/Host Selection Criteria***

**Figure 7:**
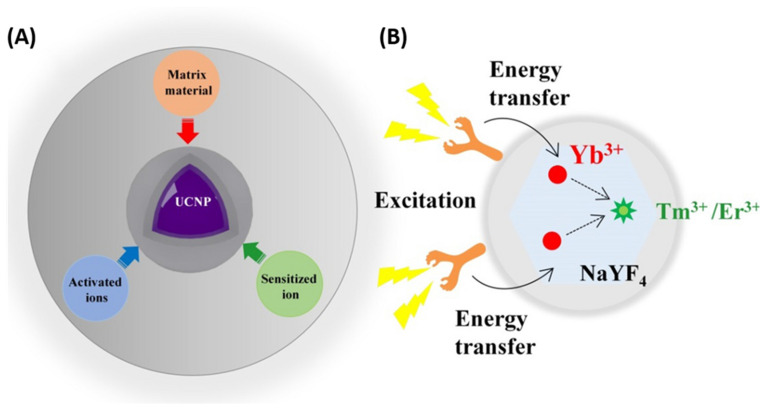
(**A**) The basic composition of UCNPs and (**B**) schematic illustration of the mechanism of organic dye-sensitized UCNPs. Reproduced with permission from Ref. [194].

The distinctive optical behavior of UCNPs is largely determined by their material composition. They typically consist of an inorganic host lattice embedded with light-absorbing sensitizer ions and light-emitting activator ions (Figure 7) [192,193,194]. Inorganic compounds based on rare earth elements—such as NaYF_4_, NaErF_4_, NaGdF_4_, NaLuGdF_4_, Y_2_O_3_, Y_2_O_2_S, and GdOCl—serve as excellent host materials due to their low phonon energy, strong chemical and thermal stability, and high optical transparency [191]. Choosing suitable host materials is crucial for synthesizing lanthanide-doped (Ln-doped) nanocrystals with targeted nanoscale features and desirable optical properties, including high upconversion efficiency and tunable emission. An ideal host should be readily synthesized with small, uniform particle size and a narrow size distribution. Additionally, low lattice phonon energy is essential, as it reduces nonradiative losses and enhances radiative emission [101]. Rare earth fluorides, particularly binary REF_3_ and ternary AREF_4_ compounds (where RE = rare earth element, A = alkali metal), are the most commonly employed host materials for UCNP fabrication [195,196,197,198,199,200,201]. Among them, hexagonal-phase NaYF_4_ nanocrystals co-doped with Yb/Er or Yb/Tm are recognized as the most efficient upconversion systems.

Activator ions serve as the primary luminescent centers in UCNPs. Among various options, trivalent lanthanide ions (Ln^3+^), particularly Er^3+^, Tm^3+^, and Ho^3+^, are most frequently used due to their stepwise energy level structures, which facilitate multiphoton absorption and efficient upconversion [74,101,202,203]. These ions also possess wide energy level gaps, which help suppress nonradiative transitions between excited states, thereby enhancing upconversion efficiency. Moreover, Yb^3+^, which shows high absorption (~980 nm, ^2^F_7/2_→^2^F_5/2_), and other rare earth elements (mainly Er^3+^, Tm^3+^, Ho^3+^, Tb^3+^, Eu^3+^, Dy^3+^, Sm^3+^, and Gd^3+^) have been successfully employed as efficient sensitizers and activators in the fabrication of UCNPs, respectively [178,191,204]. The efficiency of energy transfer in UCNPs can be enhanced by carefully tuning the inorganic host matrix along with the concentrations of sensitizer and activator ions, particularly under excitation wavelengths of 808 nm and 980 nm [205]. Importantly, the emission colors of UCNPs can be finely tuned by adjusting the concentrations of sensitizer and activator ions [206], as well as by introducing additional metal ions [111,178,207], with specific details summarized in Table 2.

Importantly, UCNPs exhibit colorful emissions, which make them promising materials for bioapplications [178]. Multiple research teams have investigated UCNPs doped with alternative lanthanide ions such as Gd^3+^, Yb^3+^, Ho^3+^, and Sm^3+^ as contrast agents for imaging modalities like MRI, CT, and PAI, aimed at enhancing the visualization of specific biological structures [216,217,218]. In recent years, the application of NaYF_4_-based UCNPs in biomedical applications has expanded rapidly, presenting promising opportunities for the advancement of novel non-invasive bioimaging techniques. Nevertheless, the search for alternative host materials for UCNPs continues in pursuit of improved performance and tailored nanoscale properties. A key limitation of NaYF_4_-based UCNPs in biological applications is the difficulty in synthesizing ultra-small nanocrystals (below 10 nm) that still exhibit strong upconversion luminescence [191]. To overcome this problem, other fluorides such as Na_x_ScF_3+x_ [219], NaYbF_4_ [220], KGdF_4_ [221], CaF_2_ [222,223], NaLuF_4_ [224,225,226], and BaLuF_5_ [227] have recently gained popularity as host materials for producing smaller UCNPs with enhanced brightness.

(iii) ***Mechanism of Upconversion—Optical Imaging Agent***

The rich energy level structures of lanthanide ions provide upconversion nanoparticles with extensive possibilities for efficient energy transfer processes, which have been thoroughly summarized and analyzed in various review articles [74,228,229]. These upconversion mechanisms can be broadly categorized into five types: (a) excited-state absorption (ESA), (b) energy transfer upconversion (ETU), (c) cooperative sensitization upconversion (CSU), (d) cross-relaxation (CR), and (e) photon avalanche (PAv) [221,230]. It is important to note that the term “upconversion” is occasionally used to refer to the overall result—namely, the conversion of long-wavelength to short-wavelength emission—and at other times to denote a specific underlying mechanism responsible for this process.

**Figure 8:**
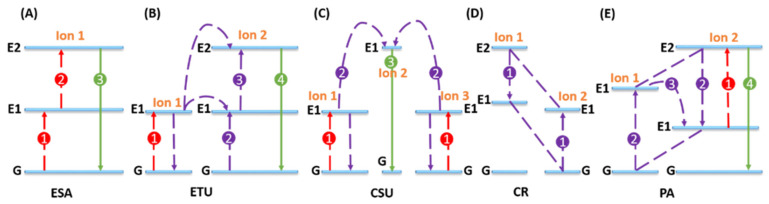
Principle of UC processes for lanthanide-doped UCNPs: (**A**) excited-state absorption (ESA), (**B**) energy transfer upconversion (ETU), (**C**) cooperative sensitization upconversion (SCU), (**D**) cross-relaxation (CR), and (**E**) photon avalanche (PAv). The red, violet, and green lines represent photon excitation, energy transfer, and emission processes, respectively.

All five mechanisms operate through the stepwise absorption of two or more photons by long-lived, metastable energy states. This process results in the population of a higher excited state, from which upconversion emission is generated. Specifically, in ESA, a single ion absorbs multiple photons in succession, with each photon promoting the ion to a higher energy level until it reaches the emissive state (Figure 8A).

ETU resembles ESA in that both involve the sequential absorption of two photons to reach a higher-energy metastable state. However, the key distinction lies in the mechanism of excitation: while ESA occurs within a single ion, ETU involves energy transfer between neighboring ions. In ETU, two adjacent ions each absorb a pump photon of the same energy, with one ion subsequently transferring its energy to the other, promoting it to the metastable level E1 (Figure 8B).

CSU, illustrated in Figure 8C, involves the interaction of three ion centers—typically with ion 1 and ion 3 being of the same type. Compared to ESA and ETU, CSU is significantly less efficient, often by several orders of magnitude, due to its reliance on quasi-virtual pair states that require higher-order quantum mechanical treatment through perturbation theory. Nevertheless, the inherently low efficiency of CSU necessitates highly localized excitation, which could be advantageous for achieving super-resolution imaging not attainable through other upconversion mechanisms.

CR, shown in Figure 8D, is a type of energy transfer arising from interactions between ions, where ion 1 transfers part of its excitation energy to ion 2 via a process involving transitions such as E2 (ion 1) + G (ion 1) + E1 (ion 2). As a fundamental ion–ion interaction mechanism, the efficiency of CR is strongly influenced by dopant concentration. While CR often leads to the well-known phenomenon of concentration quenching, where excessive dopant levels reduce emission efficiency; it can also be strategically utilized to modify emission color or to initiate efficient PAv processes.

PAv, depicted in Figure 8E, is characterized by a unique excitation mechanism that requires the pump power to exceed a specific threshold. The process begins with weak, non-resonant ground-state absorption (GSA) that populates level E1, followed by resonant excited-state absorption (ESA) that promotes electrons to the higher-energy emissive level E2. PAv is readily distinguishable by its threshold-dependent behavior, delayed luminescence onset (often taking seconds), and a steep, nonlinear increase in photoluminescence intensity near the pump threshold.

(iv) ***UCNPs works as contrast agent for PAI***

Since Alexander Graham Bell first observed the photoacoustic effect [13], significant research efforts have focused on creating efficient photoacoustic contrast agents. These include both inorganic materials—such as gold-based nanostructures, MXene derivatives, carbon-based nanomaterials, and silicon nanoparticles—and organic agents like small organic molecules, polymers, and DNA-based constructs [231,232,233,234,235]. These contrast agents have significantly broadened the scope of PAI by enhancing contrast and enabling deeper tissue imaging. Among them, upconversion nanoparticles (UCNPs) have drawn significant interest due to their ability to absorb near-infrared (NIR) light and emit visible or higher-energy photons via multi-photon upconversion processes [236]. This anti-Stokes emission reduces background interference, minimizes sample autofluorescence, and lowers the risk of tissue overheating, which is a critical advantage for in vivo imaging applications [237,238,239,240,241]. If luminescence is suppressed, either through surface quenching or hybrid material coupling, this energy is instead dissipated as localized heat, leading to transient thermoelastic expansion of the surrounding medium. This rapid expansion generates an ultrasonic pressure wave, which can be detected by photoacoustic transducers and used to reconstruct high-resolution images. These dual-functional properties position UCNPs as ideal nanoplatforms for both fluorescence and photoacoustic modalities, and as versatile tools for biological sensing and in vivo diagnostics. Representative lanthanide-based nanoprobes and their applications in PAI are summarized in Table 3.

Nevertheless, to fully realize their clinical potential, the in vivo biocompatibility and degradation behavior of UCNPs must be critically addressed.

Although UCNPs—typically composed of rare-earth doped fluoride matrices (e.g., NaYF_4_:Yb,Er/Tm)—are generally considered chemically stable and photophysically robust, their in vivo biodegradation may release lanthanide ions or fluoride species. These degradation products could potentially induce cytotoxicity, oxidative stress, or organ accumulation, especially upon prolonged exposure or high-dose administration. The cytotoxicity also leads to the rare use of bare UCNPs in medical contrast agents; thus, the application of a suitable surface modification is particularly important when it comes to the health concerns of UCNPs in vivo.

Recent research thus focuses on engineering UCNPs with biocompatible coatings (e.g., silica, PEG, phospholipids) or designing biodegradable core–shell structures to improve clearance and reduce systemic toxicity. Surface modifications not only largely improve the stability of UCNPs, but also enhance the performance of UCNPs as in vivo contrast agents by enhancing the hydrophilicity, biocompatibility, colloidal stability, and even optical intensity. It is well known that the cytotoxicity of UCNPs is a result of toxic ion (mainly fluoride ions and lanthanide ions) leakage during the degradation process. For engineered UCNPs aimed at reducing the potential degradation and increasing safety, the coating acted as a protective layer against the hydroxide ion or phosphate ion in the surrounding environment, which is corrosive to UCNPs and the cause of cytotoxic ion leakage. According to the research of Bastos et al., thick silicon-coated UCNPs have significantly lower fluoride ion leakage (4.42 µM) compare to bare UCNPs (297.37 µM) after 48 h in water [257]. The research of Saleh et al. also reported that the fluoride ion and yttrium ion leakage from thick silicon-coated UCNPs remained at a very low level (≈0 µg·mL^−1^) after over 70 h, while the fluoride ion leakage from bare UCNPs already exceeded 5000 µg·mL^−1^ in water, and the yttrium ion leakage exceeded 4000 µg·mL^−1^ at the same time [258]. As such, the safety and stability increasing effects of UCNP surface modifications were widely proved by experimental data.

Nonetheless, UCNPs with an appropriate surface modification could be excreted via the hepatobiliary excretion pathway in a shorter time period without overt tissue toxicity. This effect will be increased if coordinate with a small particle size, since smaller particles can avoid participation of intracellular catabolism and are rapidly cleared by urine. The research of Liu et al. reported that a hydrophilic polyethylene glycol (PEG)-coated 5.1 nm NaGdF_4_ UCNP sample exhibited an elimination half-life of 1.4 days after being injected into mice. The excreted particles found in the feces were confirmed by TEM image analysis to be no different in size, size distribution, and shape compared to the original particles. This suggested that PEG-coated UCNPs within living mice were not transformed [259,260]. The safety of such UCNPs can be concluded to be reliable, since the degradation time was obviously longer than the excretion time, and most particles were cleared out of the system before they had enough time to release toxic ions. Additionally, the fastest complete excretion time of NaYF_4_:Yb,Er@SiO_2_-PEI UCNPs (50 nm) is 7 days, reported by Zhang et al. [261], which was a noteworthy result in view of the particle size.

## 4. Photoacoustic Imaging—A Perfect Imaging Modality of UCNPs

Upconversion nanoprobes possess notable features such as excellent photostability, favorable biocompatibility, distinct near-infrared anti-Stokes emission, and improved tissue penetration, making them valuable tools for visualizing biological activities and detecting disease biomarkers [262,263].

### 4.1. Coated UCNPs for PAI

Lanthanide-doped upconversion nanoparticles (UCNPs) have attracted growing interest as next-generation contrast agents for photoacoustic imaging due to their chemical stability, engineerable photophysical properties, and biocompatibility. Their anti-Stokes emission, wherein low-energy near-infrared (NIR) light is absorbed and converted into higher-energy emission, offers distinct advantages for in vivo applications. This includes minimized background interference—as biological tissues lack strong anti-Stokes signals—and reduced tissue overheating, since NIR light penetrates deeply with lower absorption and scattering, lowering the risk of photothermal damage [264,265].

Although luminescence is not the primary readout in PA imaging, the balance between radiative and nonradiative relaxation pathways is central to UCNP performance. Efficient luminescence implies that the particles’ energy levels can be finely tuned. However, quenching luminescence intentionally through material design (e.g., via surface coatings or dopant optimization) allows more energy to dissipate as heat, which is crucial for generating strong PA signals.

In this context, dopant concentration, shell passivation, and hybrid construct formation are key strategies for modulating energy dissipation and optimizing PA performance.

Doping concentration significantly influences nonradiative relaxation pathways, which are crucial for thermal expansion and, consequently, PA signal generation. Sheng et al. showed that rare-earth-element doped micrometer-sized particles (e.g., NaYF_4_:Yb^3+^, Er^3+^) modified with polyacrylic acid yielded enhanced PA signals, particularly in anisotropic rod-like structures. These enhancements were attributed to optimized doping levels that promote upconversion luminescence quenching and efficient heat generation [242].

Shell passivation, such as inert NaYF_4_ coatings, plays a crucial role in reducing surface defects and suppressing surface quenching. This not only improves upconversion efficiency, but also increases the proportion of absorbed photon energy converted into heat rather than being lost via radiative emission. As shown in comparative studies, increasing shell thickness enhanced the PA signal more effectively than increasing particle size alone, confirming the pivotal role of surface engineering.

**Figure 9:**
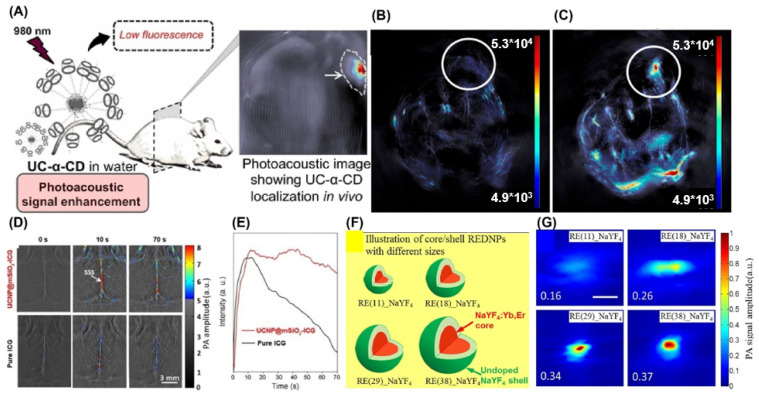
Strategies for designing lanthanide nanoparticles as photoacoustic diagnostic markers. (**A**) Diagram showing fluorescence damping followed by photoacoustic signal amplification from UCNP/α-CD dispersed in H_2_O. High-definition acoustic responses and phantom images were captured using nanosecond pulsed laser excitation at 980 nm. Reprinted with permission from Ref. [254]. PA images at the neoplasm region for mice before (**B**) and after (**C**) intratumoral injection of Nd: UCNPs@H-TiO_2_ nanocomposites, respectively. Reproduced with permission from Ref. [248]. (**D**) Mouse brain imaging was conducted following injection of UCNP@SiO_2_-ICG using 800 nm excitation with both pulsed- and continuous-wave lasers (n = 3). SSS refers to the superior sagittal sinus. (**E**) The in vivo photoacoustic intensity versus the intravenous injection time. Reproduced with permission from Ref. [246]. (**F**) Impact of nanoparticle shell thickness and size on UCNPs’ PA signals during NIR imaging. (**G**) Photoacoustic assessments were conducted with a dark-field PAM instrument with 50 MHz central frequency and confocal detection with approximately 4 ns laser pulses (975 nm) at a 10 Hz pulsing frequency. Reused with permission from Ref. [253].

Based on these insights, Zhao and Yu et al. first reported that NaYF_4_:Yb^3+^, Er^3+^ upconversion nanoparticles coated with α-cyclodextrin (UCNP/α-CD) could significantly enhance photoacoustic signals through luminescence quenching-induced nonradiative relaxation. This enhancement arises from the intrinsic heat-generating capability of UCNPs, coupled with improved thermal conductivity due to a phase transition occurring in aqueous environments. Under 980 nm laser excitation, this mechanism enabled strong PA signal output in water, allowing successful in vivo imaging of mouse kidneys (Figure 9A) [254]. These findings support the potential of UCNP/α-CD systems as effective contrast agents for diagnostic imaging applications.

As limited penetration depth is still the main disadvantage for bioimaging diagnostics, hybrid constructs that integrate UCNPs with optically absorbing materials have demonstrated dramatic improvements in PA performance. Hou et al. describe the synthesis of H-TiO_2_-decorated Nd^3+^-sensitized UCNPs using a modified Nd: YAG laser method (Nd: UCNPs@H-TiO_2_), under 808 nm excitation [248]. H-TiO_2_ not only serves as a visible-light-driven photosensitizer, but can be utilized to generate a PA signal with a high US spatial resolution determined by thermoelastic expansion. This result was confirmed experimentally for mice in comparison to the untreated tumor. Such excellent imaging performance demonstrates that Nd: UCNPs@H-TiO_2_ serves as an obvious contrast agent in optical absorption between exogenous and endogenous environments to overcome the limitation of fundamental penetration depth (Figure 9B,C) [248]. Meanwhile, the particle size, morphology, and coating are all being explored to optimize UCNPs for PAI applications. Lv et al. designed a novel contrast agent that can work well in their newly designed nanoplatform for PAI. The material consists of a UCNP core–shell structure with an additional mesoporous silica loaded with indocyanine green (ICG) molecule (UCNP@mSiO_2_-ICG). Compared with pure ICG, sealing the ICG within a mesoporous silica exhibited significantly elevated PA signals due to ICG’s strong NIR absorption and improved stability from silica encapsulation. These constructs not only boosted imaging depth (up to 1.5 cm in vivo), but also mitigated dye leakage and photobleaching (Figure 9D) [246]. Meanwhile, Sheng et al. [253] reported that rare-earth-element-doped micrometer-sized particles can be used for PAI optimized contrast visualization. The authors showed that the morphology, particle size, and proper coating have a major impact on influencing the PA signal intensity, especially emphasizing that coating an inert NaYF_4_ shell can enhance luminescent emission more significantly than increasing the particle size, indicating that coating an inert shell can effectively reduce the surface quenching effects (Figure 9F,G).

Together, these advances underscore the critical relationship between nanoparticle design and PA imaging performance, offering pathways to optimize contrast agent efficacy for deep-tissue diagnostics and real-time biomedical applications.

### 4.2. UCNP Nanocomposites for PAI

**Figure 10:**
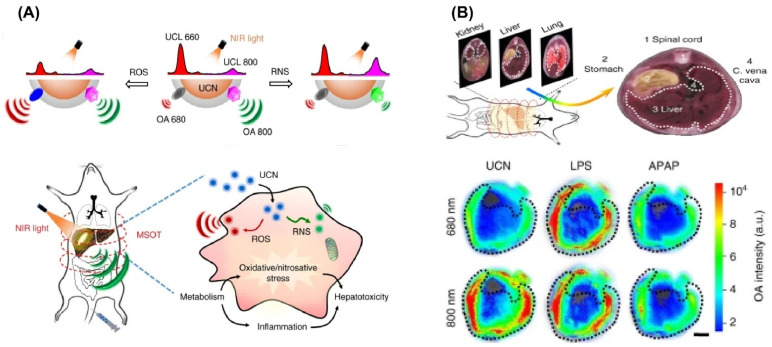
Schematic representation of UCNP-based responsive nanoprobes tailored for photoacoustic bioimaging applications. (**A**) UCNP–dye composite nanoprobes designed for monitoring dynamic correlations of reactive oxygen species (ROS) and reactive nitrogen species (RNS) in liver pathology. (**B**) Multispectral optoacoustic tomography (MSOT) images at 680 nm and 800 nm showing regions of interest (ROIs) in liver cross-sections with pseudo-color mapping after nanoprobe injection in models treated with lipopolysaccharide (LPS) and acetaminophen (APA). Reproduced with permission from Ref. [267].

Whilst the findings discussed above highlight the potential of UCNPs as PAI contrast agents, their effectiveness in deep-tissue diagnostics remains limited due to relatively low photoacoustic conversion efficiency and interference from non-specific background signals [266]. To address these limitations, Zhuang et al. developed ONOO^−^-responsive UCY7 nanoprobes, constructed by coordinating heptamethine cyanine dyes with UCNPs. These nanoprobes demonstrated excellent sensitivity, selectivity, photostability, and biosafety for both in vitro and in vivo detection of ONOO^−^. By integrating the dye with UCNPs, they effectively mitigated the photobleaching issues of cyanine dyes and enhanced tissue penetration by converting visible 660 nm light from P-cy7 into 980 nm NIR light absorbed by UCY7. This strategy led to superior in vitro PAI performance and enabled real-time imaging of liver injury in living subjects [245]. In addition, Xing and Gao et al. developed UCNP–cyanine dye nanocomposites to enable PAI of dynamic redox changes during disease progression (Figure 10) [267].

**Figure 11:**
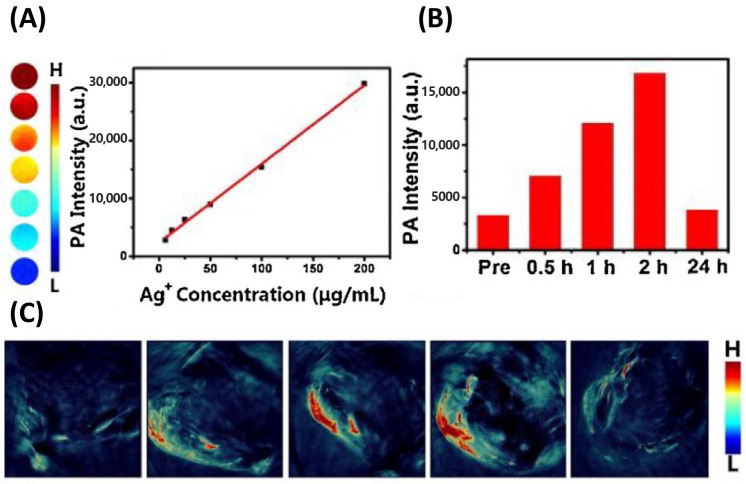
Multimodal imaging performance of UCNP@CS@Ag_2_Se nanocomposites in vitro and in vivo. (**A**) PA signal intensity of UCNP@CS@Ag_2_Se nanocomposites at varying concentrations in vitro, (**B**) PA signal response showing concentration dependence, and (**C**) time-course PA imaging of pre- and post-injection assessment in mice with tumors of UCNP@CS@Ag_2_Se nanocomposites. Reproduced with permission from Ref. [250].

Du et al. developed an innovative multifunctional nanoplatform by in situ synthesizing ultrasmall Ag_2_Se nanodots uniformly anchored onto UCNP surfaces via an intermediate CS shell layer (Figure 11). The PA signal gradually increased within 2 h of injection of UCNP@CS@Ag_2_Se nanocomposites, suggesting that a significant amount of nanocomposites preferentially accumulate at the tumor site. Quantitative analysis revealed that the PA signal within the tumor increased five-fold two hours after injection, which is probably owed to the enhanced permeability and retention (EPR) effect occurring during systemic circulation [250].

### 4.3. Outcomes of Combining UCNPs for PAI with Other Imaging Modalities

**Figure 12:**
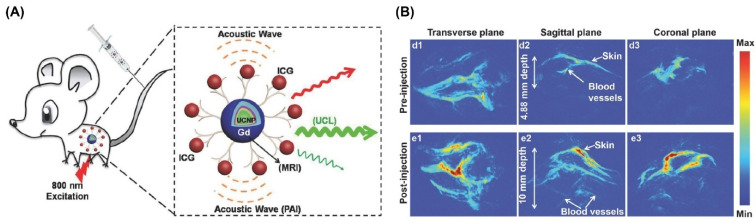
(**A**) Schematic of deep-tissue imaging utilizing high-performance multi-shell UCNPs for in vivo PAI, UCL, and MRI. The layered structure enhances PAI penetration depth, supporting applications such as early tumor detection, drug tracking, and surgical navigation. (**B**) Photoacoustic images showing tumor and vasculature visualization in mice post-intravenous nanoprobe injection. Reproduced with permission from Ref. [243].

Imaging technologies have greatly advanced our ability to observe biochemical processes and understand the relationship between anatomical structure and biological role at both cellular and anatomical levels. Nonetheless, each imaging modality comes with inherent drawbacks—such as limited sensitivity or resolution—which can hinder the acquisition of precise and dependable data from target tissues. A practical solution to these challenges is the integration of multiple imaging techniques to boost spatial resolution and signal fidelity performance. In particular, combining luminescence imaging with photoacoustic imaging (PAI) offers a cost-effective, intraoperative platform that is well-suited for surgical navigation and enables high-resolution visualization of deep-tissue structures. For instance, the material N-hydroxysuccinimide-labeled indocyanine green (ICG) immobilizing on the surface of pre-fabricated layered UCNPs enabled simultaneous UCL, PAI (Figure 12), and magnetic resonance imaging (MRI) in vivo. Attaching ICG to the UCNP surface significantly boosts UCL intensity under 800 nm excitation, owing to efficient energy transfer. Meanwhile, this unique design increases the imaging depth (from 4.8 mm up to 10 mm) of PAI, and the signal can be increased up to 1.8-fold 6 h post-injection. This effect primarily results from the EPR phenomenon in leaky tumor vasculature combined with cellular uptake via endocytosis. Following this, combination of modalities can provide much more detailed information for clinical diagnosis [243]. In addition, both upconversion luminescence (UCL) and photoacoustic (PA) tumor imaging exhibited excellent biocompatibility and high specificity in sensing, offering promising prospects for deep-tissue multimodal imaging and integrated phototherapeutic applications.

**Figure 13:**
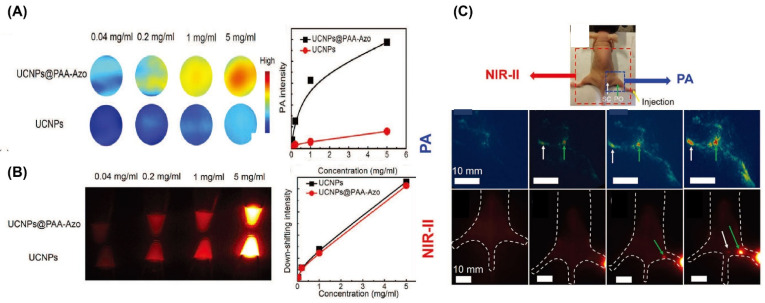
(**A**) PA imaging and related signal intensities of UCNPs and UCNPs@PAA-Azo across a series of concentrations (0.04, 0.2, 1.0, and 5.0 mg/mL), demonstrating a concentration-dependent enhancement. (**B**) NIR-II fluorescence (1350 nm, 300 ms exposure) and signal comparison between UCNPs and UCNPs@PAA-Azo in vials. (**C**) Dual-modality imaging of lymph nodes (LNs) in mice using UCNPs@PAA-Azo: photograph showing NIR-II (red) and PA (blue) imaging zones; dynamic PAI and NIR-II imaging of sacral (SC) and popliteal (PO) LNs post-injection (0, 10, 30, 60, 120 min); guided surgical removal of LNs under NIR-II imaging. Reproduced with permission from Ref. [249].

He et al. introduced a method utilizing UCNPs coated with a photo-switchable azobenzene-containing polymer (PAA-Azo), which enhanced PA signals up to six-fold compared to unmodified UCNPs while maintaining stable NIR-II emission. Nevertheless, the relatively long acquisition time (approximately 5 min) limits the real-time applicability of PAI. Integrating PA imaging with NIR-II imaging within a single platform offers a promising approach to enhance diagnostic precision and efficiency (Figure 13) [249]. Additionally, Sheng et al. demonstrated that PAA-coated REDP nanorods show significant potential as dual-modality agents for luminescence and PAI [242].

**Figure 14:**
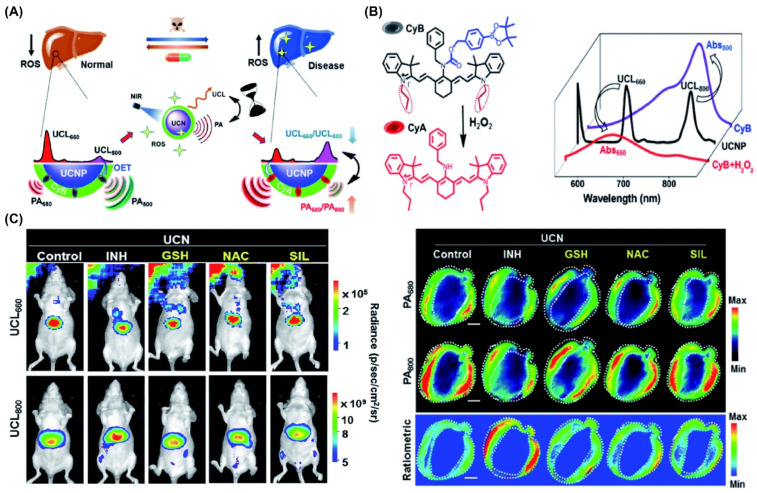
Multi-platform lanthanide nanoprobes for combined integrated luminescence and photoacoustic approaches to liver pathology visualization. (**A**) Diagram of UCL/PA dual-modality probes developed for concurrent tracking of liver disease progression and therapy evaluation. (**B**,**C**) In vivo ratiometric imaging via UCL and PA techniques to monitor liver injury caused by medication and antioxidant treatment outcomes. Reproduced with permission from Ref. [244].

Although multimodal optical imaging techniques have facilitated the visualization of significant pathological changes in living organisms, most still struggle to dynamically monitor bioindicator responses. This limitation largely stems from challenges in designing stimuli-responsive, multimodal imaging probes. To address this, Xing et al. developed a technology combining deep-tissue-penetrating multispectral optoacoustic tomography (MSOT) with upconversion luminescence (UCL) imaging by integrating reactive oxygen species (ROS)-sensitive chromophores with UCNPs (Figure 14) [244]. The interaction between pathological ROS such as H_2_O_2_ and the nanoprobes induces a shift in absorption, detectable through ratiometric MSOT signals. This system offers a self-calibrated cross-referencing approach, maximizing both sensitivity and specificity. Utilizing this combined PA and UCL imaging method, the authors successfully enabled precise monitoring of the intricate link between redox imbalance and hepatic disorders, proving valuable for in vivo evaluation of hepatoprotective therapies. (Figure 14).

**Figure 15:**
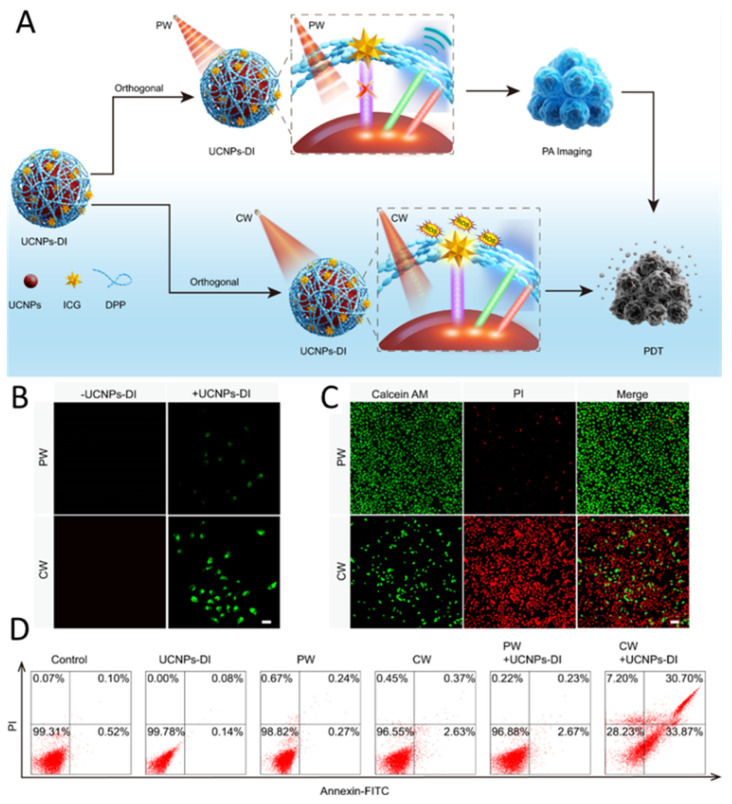
Design and evaluation of orthogonal UCNPs-DI nanoagent. (**A**) Illustration of UCNPs-DI enabling photoacoustic imaging-guided, on-demand therapy. (**B**) Intracellular 1O_2_ generation in SOS G-stained MCF7 cells under various treatments. Scale bar: 50 μm. (**C**) Confocal fluorescence images of MCF7 cells co-stained with calcein AM (green, live) and propidium iodide (PI, red, dead) following different treatments. Scale bar: 100 μm. (**D**) Flow cytometric evaluation of MCF7 cell viability under different treatment conditions. Reprinted with permission from Ref. [268].

In a separate study, Zhang et al. presented a straightforward strategy to develop an upconversion-based nanoagent (UCNPs-DI) by attaching a diketopyrrolopyrrole (DPP) polymer dye to the nanoparticle surface. This dye exhibited strong absorption overlapping with the visible emission of the UCNPs, while also incorporating indocyanine green (ICG) as a photosensitizer responsive to near-infrared (NIR) light. Under pulsed-wave (PW) 980 nm laser excitation, ICG remains inactive, while the visible light emitted by UCNPs is absorbed by DPP to produce strong photoacoustic signals without inducing photothermal or photodynamic effects. In contrast, UCNPs, DPP, free ICG, and UCNPs-I generated only weak signals. These findings demonstrate that UCNP-DI enables enhanced, long-lasting, real-time PA imaging with minimal phototoxicity, facilitating effective PA-guided phototherapy. Additionally, altering the 980 nm laser from PW to continuous-wave (CW) mode significantly boosts its photodynamic therapy effect [268] (Figure 15).

**Figure 16:**
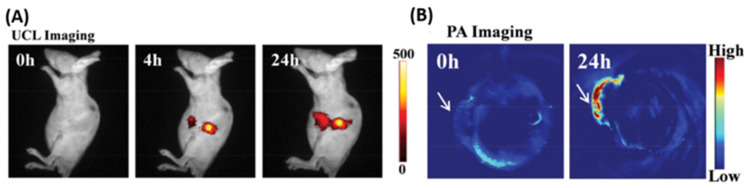
In vivo multimodal imaging. (**A**) UCL imaging, (**B**) photoacoustic imaging of HeLa tumor-bearing mice captured at various time intervals after i.v. injection with nanorod dimer-UCNP-Ce6 (200 µL 2 mg·mL^−1^, in terms of the nanorod amount). The colors in the diagram represent the strength of the signal. Reprinted with permission from Ref. [252].

Sun et al. developed a new system using UCNPs in connection with a mixture of thiolated oligonucleotides and heterobifunctional polymer molecules grafted onto nanorod dimers. The synthesized nanorod dimer-UCNP-Ce6 exhibited a notably high efficiency in UCL-PA multimodal imaging systems. Figure 13 confirmed that PAI was effectively attributed to the material’s strong NIR absorbance. Photoacoustic imaging conducted 24 h after contrast agent administration revealed a markedly enhanced signal across the entire tumor area. This enabled clear visualization of tumor depth and structural details, highlighting the potential of DNA-guided multifunctional nanoagent systems for future clinical applications in cancer diagnosis and treatment [252] (Figure 16).

**Figure 17:**
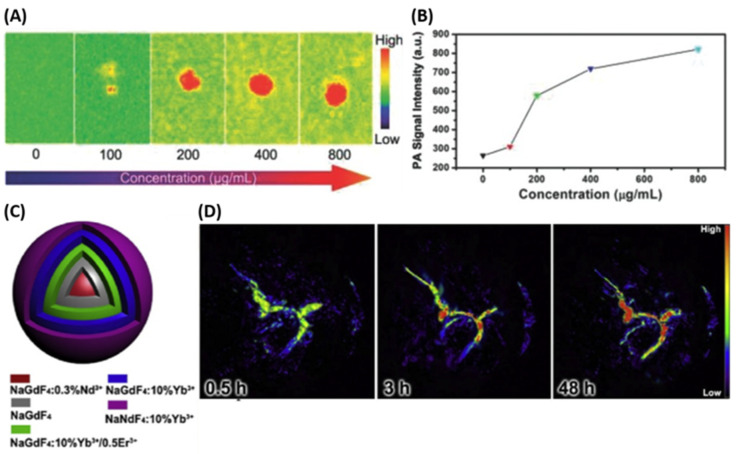
(**A**,**B**) PA signal intensity of UCNPs@MS-Au_25_-PEG decreases with dilution in distilled water. (**C**) Diagram showing the structure of the designed LDNPs-5. (**D**) In vivo PA imaging of tumor sites at different times after intravenous injection of LDNPs-5-Au_25_-PEG. Reproduced with permission from Ref. [247] and Ref. [251].

Currently, many contrast agents have been developed to support not only dual-, but also tri- and tetra-modal medical imaging. For instance, He et al. synthesized UCNPs@MS-Au25-PEG materials for PA imaging, demonstrating clear photoacoustic responses in an experiment involving concentration gradients under 808 nm laser excitation (Figure 17A,B). This single NIR light-triggered tri-modal imaging capability enables in vivo bioimaging and imaging-guided malignancy therapy. The presence of nanoparticles can be further validated by PAI, which offers high-resolution, non-invasive visualization of tissue structures, making it well-suited for diagnostics. Notably, the PA signal intensity at neoplasm sites increases over time following injection, showcasing excellent PAI performance. Moreover, PAI provides detailed structural information important for cancer detection and therapy [247]. Additionally, He et al. [251] successfully synthesized NaGdF_4_:0.3%Nd@-, NaGdF_4_@-, NaGdF_4_:10%Yb/1%Er@-, NaGdF_4_:10%Yb@-, and NaGdF_4_:10%Yb-(LDNPs-5)-Au_25_-PEG multi-core–shell structures. This integrated platform enables both energy-blocking and synergistic enhancement through the combination of photoacoustic imaging (PAI), fluorescence imaging (FI), and photothermal imaging (PTI), demonstrating the effectiveness of multimodal imaging for visualizing drug delivery processes (Figure 17C,D).

## 5. Outlook and Future Developments

Upconversion nanoparticles (UCNPs) have emerged as a promising class of contrast agents for photoacoustic imaging (PAI), offering distinct advantages over conventional dyes and metallic nanoparticles. Their unique optical properties, including narrow emission bands, near-infrared (NIR) excitation capabilities, and deep-tissue penetration, minimize background interference while enabling high-resolution imaging. Additionally, UCNPs possess inherent luminescence that facilitates dual-modal imaging when combined with PAI, allowing cross-validation of signals. The ease of surface functionalization further enhances their utility, as targeting ligands such as antibodies or peptides can be conjugated for precise subcellular imaging and theranostic applications. These features make UCNPs particularly valuable in preclinical research, where they have demonstrated excellent performance in tumor imaging, vascular mapping, and drug delivery monitoring in animal models [269,270].

Despite these advantages, significant challenges must be addressed before UCNPs can transition from laboratory research to widespread clinical use in diagnostics. One major limitation is biocompatibility, as certain UCNP compositions may trigger inflammatory responses or exhibit long-term accumulation in organs such as the liver and spleen. Furthermore, the relatively high doses required to generate detectable photoacoustic signals raise concerns about potential toxicity in human applications. Targeting efficiency in vivo remains another hurdle, with studies showing less than 5% tumor uptake in some cases due to nonspecific binding and clearance by the reticuloendothelial system. These biological challenges are compounded by technical and regulatory barriers, including the lack of standardized protocols for toxicity assessment and the high costs associated with scalable synthesis and purification compared to clinically approved contrast agents like indocyanine green (ICG).

To bridge this gap between preclinical success and clinical adoption, future research should prioritize three key areas. First, optimizing biocompatibility through innovative surface coatings—such as biodegradable silica or phospholipid layers—could mitigate immune responses while maintaining optical performance. Second, enhancing functional performance by engineering UCNPs with higher absorption coefficients in the NIR-II window (1000–1700 nm) would improve signal sensitivity at lower doses. Active targeting strategies, such as pH-responsive ligands or tumor microenvironment-specific probes, could further boost specificity. Finally, concerted efforts toward clinical integration are essential. This includes validating UCNP-based PAI in large-animal models to assess scalability and combining UCNP-enhanced molecular imaging with existing clinical ultrasound systems to leverage established infrastructure.

While UCNPs may not replace conventional imaging modalities like MRI or CT, their unique capabilities position them as powerful complementary tools. By addressing current limitations in biocompatibility, targeting efficiency, and regulatory readiness, UCNPs have the potential to transition from research curiosities to clinically viable contrast agents. As photoacoustic imaging technology advances and our understanding of nanomaterial design deepens, UCNPs could play a pivotal role in unlocking new diagnostic and theranostic applications—ultimately enhancing the precision and functionality of medical imaging.

## Figures and Tables

**Table 1:** Main synthetic methods for UCNP preparation and their advantages and disadvantages.

Main Synthetic Method	Material, Refs	Size Range (nm)	Advantages	Disadvantages
Thermal decomposition	NaNdF_4_ [180] β-NaErF_4_ [181]	50–500	High-quality, uniform size	Intermediate toxicity, high cost
Microemulsion	NaYF_4_ [182] LaF_3_ [183]	4–500	Easy to operate, narrow size distribution, high stability	Calcination or annealing usually required
Phase transfer hydrothermal synthesis	(La-Dy)VO_4_ [184] YVO_4_ [184] NaYF_4_ [185]	10–1000	Good dispersion, simple procedures, tunable size	Specialized reaction vessels are needed
Sol-gel processing	GdVO_4_ [186]	30–600	Cheap raw materials, simple procedures	Broad particle size and unsuitable for bioapplication

**Table 2:** The doping ratio of different ions of UCNPs as contrast agents working in PAI and their major emission wavelengths.

Dopant Ions and Composition				Major λ Emissions (nm)			Reference
Host Lattice	Sensitizer	Activator	Shell	Blue	Green	Red	
β-NaYF_4_	20%Yb^3+^	2%Er^3+^		450, 476	520	654	Ref. [208]
	20%Yb^3+^	0.2%Tm^3+^			540		Ref. [209]
	20%Yb^3+^	2%Ho^3+^			541		Ref. [209]
β-NaYF_4_	20%Yb^3+^	0.3%Tm^3+^	20%Yb^3+^, 2%Er^3+^	450	520	653	Ref. [210]
				475	540		
Li^+^ doped β-NaYF_4_	20%Yb^3+^	0.5%Tm^3+^		452, 479		650	Ref. [211]
Mn^+^ doped β-NaYF_4_	20%Yb^3+^	2%Er^3+^				657	Ref. [212]
β-NaYF_4_	18.6%Yb^3+^	2.2%Er^3+^	TRITC-SiO_2_	407			Ref. [213]
	25%Yb^3+^	0.3%Tm^3+^	SiO_2_	450, 479	521, 539	651	Ref. [85]
	25%Yb^3+^	0.3%Tm^3+^	FITC-SiO_2_	450, 479	521, 539, 580	651	Ref. [214]
α-NaYF_4_	25%Yb^3+^	0.3%Tm^3+^	QD-SiO_2_	450, 479	540	651	Ref. [214]
	20%Yb^3+^	2%Er^3+^		411		660	Ref. [202]
	20%Yb^3+^	0.2%Tm^3+^		450	540	644	Ref. [215]
	20%Yb^3+^	0.2%Er^3+^		475	525	693	Ref. [215]

**Table 3:** Summary of representative UCNPs applied for photoacoustic imaging.

Photoacoustic Contrast Agent	Excitation λ (nm)	PA Sensitivity	Size (nm)	Multimodal Imaging Capability	In Vivo/In Vitro	Reference
PAA-NaYF_4_:Yb,Er	520, 975	-	40–60	PAI	In vivo	Ref. [242]
NaYF_4_:Yb:Er@NaYF_4_:Yb@NaNdF_4_:Yb@NaYF_4_ @NaGdF_4_-HAD-G_2_ ICG (CS2-ICG)	808/540, 650	ICG	54.3 ± 4.1	MRI, PA, UCL	both	Ref. [243]
CyB/NaGdF_4_:Yb/Tm/Er@NaGdF_4_	980/650, 800	Isoniazid (INH)	~78	PAI, UCL	In vivo	Ref. [244]
UCY7	680	ONOO^−^	~35	PAI, RFLI	In vivo	Ref. [245]
UCNP@mSiO_2_-ICG	800	ICG	~60	PA, UCL	both	Ref. [246]
UCNPs@MS-Au_25_ -PEG	808		28–46	PA, MRI, CT, PDT, PTT	both	Ref. [247]
Nd:UCNPs@H-TiO_2_	808	H-TiO_2_	~60	PA, PDT, PTT, UCL	both	Ref. [248]
UCNP@PAA-AzoNaYF_4_:Yb30%, Tm0.5%, Nd5%	808	Azobenzene-containing poly	~21	PA, NIR-II	both	Ref. [249]
UCNPs@CS#Ag_2_Se	808	Ag^+^	141.8	UCL, CT, PAI, PTT	both	Ref. [250]
LDNPs-Au_25_-PEG	808		~40	PTT, PA, PTI, PDT, MRI	both	Ref. [251]
Nanorod (NR) dimer-UCNP-Ce_6_	980, 808	NR-dimer	~70	PA, UCL, CT, MRI	In vivo	Ref. [252]
Core/Shell β-NaYF_4_:Yb,Er	975		50.3 ± 5.6	PAT	both	Ref. [253]
NaYF_4_:Yb/Er@ α-cyclodextrin	980	α-CD		PAI	In vivo	Ref. [254]
**PoP-UCNP**	980	PoP combined with detergent	74 ± 3.6	PA, FL, UC, PET, CL, CT	In vivo	Ref. [255]
**LDNP@DMSN-Au@CaCO_3_**	980	Au-NPs	~174	PA, NIR-II, FL	both	Ref. [256]

FL, fluorescence; UC, NIR-to-NIR upconversion (UC) luminescence; PET, positron emission tomography; CL, Cerenkov luminescence; CT, computed tomography; RFLI, ratio-fluorescent imaging; NIR-II, second near-infrared; MRI, magnetic resonance imaging; PDT, photodynamic therapy; PTT, photothermal therapy; UCL, upconversion luminescence; PAT, photoacoustic tomography; PTI, photothermal imaging.
